# Association between serum uric acid levels and mortality: a nationwide community-based cohort study

**DOI:** 10.1038/s41598-020-63134-0

**Published:** 2020-04-08

**Authors:** Tsuneo Konta, Kazunobu Ichikawa, Ryo Kawasaki, Shouichi Fujimoto, Kunitoshi Iseki, Toshiki Moriyama, Kunihiro Yamagata, Kazuhiko Tsuruya, Ichiei Narita, Masahide Kondo, Yugo Shibagaki, Masato Kasahara, Koichi Asahi, Tsuyoshi Watanabe

**Affiliations:** 1 Department of Public Health and Hygiene, Yamagata University School of Medicine, Yamagata, Japan; 2 Department of Cardiology, Pulmonology, and Nephrology, Yamagata University School of Medicine, Yamagata, Japan; 3 Department of Vision Informatics (Topcon), Osaka University Graduate School of Medicine, Osaka, Japan; 4 Steering Committee of Research on Design of the Comprehensive Health Care System for Chronic Kidney Disease (CKD) Based on the Individual Risk Assessment by Specific Health Checkup, Fukushima, Japan

**Keywords:** Predictive markers, Epidemiology, Epidemiology, Risk factors

## Abstract

Hyperuricemia is associated with all-cause and cardiovascular mortality. However, the threshold value of serum uric acid levels for increased risk of mortality has not been determined. This large-scale cohort study used a nationwide database of 500,511 Japanese subjects (40–74 years) who participated in the annual health checkup and were followed up for 7 years. The association of serum uric acid levels at baseline with cardiovascular and all-cause mortality was examined. The Cox proportional hazard model analysis with adjustment for possible confounders revealed that the all-cause and cardiovascular mortality showed a J-shaped association with serum uric acid levels at baseline in both men and women. A significant increase in the hazard ratio for all-cause mortality was noted with serum uric acid levels ≥ 7 mg/dL in men and ≥ 5 mg/dL in women. A similar trend was observed for cardiovascular mortality. This study disclosed that even a slight increase in serum uric acid levels was an independent risk factor for all-cause and cardiovascular mortality in both men and women in a community-based population. Moreover, the threshold values of uric acid for mortality might be different for men and women.

## Introduction

Hyperuricemia is an established risk factor for gout and end-stage kidney disease^1^. Furthermore, various studies have suggested the association of hyperuricemia with all-cause and cardiovascular mortality^2–9^. However, the definition of hyperuricemia varies in each study; therefore, the threshold value of serum uric acid for increased risk of mortality, which is essential for the initiation of treatment, has not been determined.

When examining the association between uric acid levels and prognosis, several points must be considered. First, hyperuricemia is often accompanied by other risk factors such as hypertension, obesity, diabetes, dyslipidemia, and renal insufficiency. This makes it difficult to determine whether hyperuricemia is an independent risk factor or a mere bystander with the other risk factors. Second, sex differences in the distribution of serum uric acid levels exist, and this may modify the association between serum uric acid levels and mortality^10–12^. Third, some studies showed that the association between serum uric levels and mortality was not linear, but J-shaped^11–13^, indicating that the conventional classification of uric acid levels, such as hyperuricemia (e.g., > 7 mg/dL) or per 1 mg/dL increase, might be inappropriate to evaluate the risk of increased serum uric acid levels on mortality.

Considering these conditions, a large-scale study with a sufficient number of events, various correction factors, and a sex-specific reference range of uric acid is necessary to address this issue. However, in most previous studies, the conventional classification or quartile/quintile subgrouping of uric acid was used, and the number of events was insufficient to perform a multivariate analysis using a fine classification of uric acid levels. Accordingly, the association between uric acid levels and mortality remains unclear.

To clarify the threshold values of uric acid levels for increased risk for all-cause and cardiovascular mortality, this study retrieved a database of 500,511 community-based Japanese participants with 5,578 mortality events from a nationwide prospective cohort study.

## Methods

### Study population

This study was part of an ongoing “Research on design of the comprehensive health care system for chronic kidney disease (CKD) based on individual risk assessment by Specific Health Checkup” study. The Specific Health Check and Guidance is an annual health checkup for all inhabitants of Japan between 40 and 74 years and is covered by the Japanese national health insurance. We utilized databases obtained from 7 areas: Fukushima, Niigata, Ibaraki, Toyonaka, Fukuoka, Miyazaki, and Okinawa. Data were collected from 664,926 subjects (male: 284,320; female: 380,606; age: 40–74 years) who participated in the baseline health checkups between 2008 and 2014. The study was conducted in accordance with the guideline of the Declaration of Helsinki and was granted ethical approval by the ethics committees of Yamagata University (Approval No. 2008-103). The ethics committees of Yamagata University waived the need for informed consent from each participant because all data were anonymized before analysis. The details of this study have been described elsewhere^14,15^.

Among the 664,926 participants, 164,415 participants (male: 68,592; female: 95,823) were excluded from this study because some essential data, including serum uric acid levels, were incomplete. Therefore, data from 500,511 subjects (male: 215,728; female: 284,783; age: 40–74 years) were included in this analysis. The association between serum uric acid levels at baseline and all-cause and cardiovascular mortality during the 7-year follow-up period (median 3.6 years) was examined.

### Measurements

The subjects completed a self-report questionnaire to document their medical history, current medications, smoking habits (smoker or non-smoker), and alcohol consumption (drinker or non-drinker). Systolic (SBP) and diastolic blood pressure were measured using a standard sphygmomanometer or an automated device, with subjects in a sitting position. Body mass index (BMI) was calculated as weight in kilograms (kg) divided by the square of the height (m^2^). Plasma glucose levels were mainly measured using the hexokinase enzymatic reference method. Triglyceride and low-density lipoprotein cholesterol (LDL-C) concentrations were measured using enzymatic methods, and high-density lipoprotein cholesterol (HDL-C) concentrations were measured directly. Serum uric acid levels were mainly measured using an enzymatic method, and hyperuricemia was defined as serum uric acid levels >7 mg/dL. Serum creatinine was measured by an enzymatic method, and the estimated glomerular filtration rate (eGFR) was obtained using the Japanese equation^16^. All blood and urine analyses were performed in local laboratories. The methods for these analyses were not calibrated between laboratories; however, the analyses were performed according to the Japan Society of Clinical Chemistry recommended methods for laboratory tests, which have been widely adopted by laboratories across Japan. The measured items and the disease criteria in this study are based on the methods used in our previous studies^10,14^.

The incidence of death was monitored annually at the end of 2015, and the causes of death were determined by reviewing death certificates until 2015. Death certificates of the deceased participants were collected with permission from the Management and Coordination Agency of the Japanese government. The death code (International Classification of Diseases, 10th Revision) and date of death were reviewed.

### Statistical analysis

The chi-squared test was used to evaluate differences in proportions. To compare the mean values among the groups, a one-way analysis of variance was conducted. To examine the factors related to mortality, Kaplan–Meier and multivariate Cox proportional hazard analyses were performed. Moreover, adjustment for possible confounding factors such as age, smoking habits, alcohol consumption, eGFR, BMI, SBP, HbA1c (NGSP), triglyceride levels, LDL-C and HDL-C levels, and the use of antihypertensive, anti-diabetic, and lipid-lowering medications was performed. The group with the lowest hazard ratio (HR) for mortality was used as a reference. To confirm the association, a sensitivity analysis was performed in 428,347 subjects with preserved renal function (eGFR ≥ 60 mL/min/1.73 m^2^) and in 349,906 subjects not taking any antihypertensive medications. Continuous data are expressed as the mean ± standard deviation. All statistical analyses were performed using the JMP version 10 software (SAS Institute Inc., Cary, NC, USA) and Stata version 14 software (Stata Corp LP, College Station, TX, USA). A *P* value < 0.05 was considered statistically significant.

## Results

The subjects’ clinical characteristics at baseline are presented in Table 1. The mean age was 62 years in both men and women, and the mean serum uric acid level was 6.1 mg/dL and 4.7 mg/dL, respectively. Both male and female subjects with higher serum uric acid levels were more likely to be taking antihypertensive and lipid-lowering medications, and to show a higher prevalence of smoking and alcohol consumption, higher values of BMI, SBP, triglyceride, and LDL-C, and lower values of eGFR and HDL-C. Among subjects with higher serum uric acid levels, the mean age, HbA1c, and prevalence of anti-diabetic medication were lower in men than in women.

**Table 1:** Baseline characteristics of the study population.

Men	Total	Serum uric acid (mg/dL)								P value
		≤ 2.9	3.0-3.9	4.0-4.9	5.0-5.9	6.0-6.9	7.0-7.9	8.0-8.9	> 9.0	
Number	215,728	1,992	9,449	30,383	60,311	61,317	34,764	12,605	4,907	
Age (years)	62.2 ± 9.2	63.2 ± 8.8	63.8 ± 8.3	63.3 ± 8.5	62.7 ± 8.9	62.0 ± 9.3	61.3 ± 9.5	60.2 ± 9.7	59.4 ± 9.9	<0.01
Smoker (%)	27.3	28.4	27.7	27.8	27.1	26.8	27.0	28.6	32.1	<0.01
Alcohol consumption (%)	69.9	60.6	64.1	64.6	67.6	71.5	74.3	77.4	79.7	<0.01
Body Mass Index (kg/m^2^)	24.0 ± 3.2	23.0 ± 3.3	23.0 ± 3.2	23.1 ± 3.1	23.6 ± 3.1	24.2 ± 3.1	24.8 ± 3.3	25.2 ± 3.5	25.3 ± 3.7	<0.01
eGFR (ml/min/1.73m^2^)	74.9 ± 17.7	84.8 ± 23.9	82.8 ± 24.6	80.2 ± 17.5	76.9 ± 16.7	73.5 ± 16.2	70.5 ± 16.8	68.1 ± 16.7	65.0 ± 18.6	<0.01
Systolic blood pressure (mmHg)	130.9 ± 17.2	128.4 ± 17.1	129.4 ± 17.1	129.2 ± 17.3	129.9 ± 17.1	131.2 ± 16.9	132.6 ± 17.1	134.0 ± 17.6	134.5 ± 18.3	<0.01
HbA1c (NGSP) (%)	5.8 ± 0.9	6.2 ± 1.6	6.1 ± 1.4	5.9 ± 1.2	5.8 ± 0.9	5.7 ± 0.7	5.7 ± 0.7	5.7 ± 0.6	5.7 ± 0.7	<0.01
Triglycerides (mg/dL)	143.5 ± 112.7	119.4 ± 85.5	119.2 ± 90.2	122.2 ± 94.8	129.5 ± 91.7	144.5 ± 105.3	164.8 ± 129.4	189.9 ± 157.1	221.1 ± 198.4	<0.01
HDL-cholesterol (mg/dL)	56.5 ± 15.2	58.8 ± 16.1	58.6 ± 15.6	58.2 ± 15.7	57.1 ± 15.0	56.1 ± 14.9	55.0 ± 15.1	54.3 ± 14.8	53.5 ± 14.9	<0.01
LDL-cholesterol (mg/dL)	120.3 ± 31.0	114.1 ± 30.4	116.3 ± 29.8	117.6 ± 29.9	119.8 ± 29.8	121.3 ± 30.6	122.3 ± 32.2	122.6 ± 34.1	120.2 ± 37.8	<0.01
Antihypertensive medication (%)	33.3	28.5	29.2	28.8	30.6	35.2	37.8	36.8	38.5	<0.01
Antidiabetic medication (%)	8.7	12.1	11.9	10.6	7.9	6.7	6.0	5.2	5.7	<0.01
Lipid lowering medication (%)	10.8	12.1	11.2	10.9	10.8	11.1	10.8	9.2	9.1	<0.01
**Women**		**Serum uric acid (mg/dL)**								**P value**
	**Total**	**≤ 2**.**9**	**3**.**0-3**.**9**	**4**.**0-4**.**9**	**5**.**0-5**.**9**	**6**.**0-6**.**9**	**7**.**0-7**.**9**	**8**.**0-**	**> 9**.**0**	
Number	284,783	11,753	59,448	107,025	72,205	25,762	6,625	1,474	491	
Age (years)	62.4 ± 8.7	61.0 ± 9.8	61.3 ± 9.4	62.2 ± 8.7	63.2 ± 8.1	63.7 ± 7.8	63.7 ± 7.9	64.5 ± 7.8	63.9 ± 8.4	<0.01
Smoker (%)	6.5	6.6	6.2	6.0	6.7	7.8	9.7	10.7	10.6	<0.01
Alcohol consumption (%)	28.9	25.7	26.5	28.8	30.3	31.1	32.5	33.7	28.2	<0.01
Body Mass Index (kg/m^2^)	23.0 ± 3.6	21.6 ± 3.2	21.8 ± 3.1	22.6 ± 3.3	23.8 ± 3.7	25.0 ± 4.0	25.9 ± 4.4	26.1 ± 4.5	25.8 ± 5.1	<0.01
eGFR (ml/min/1.73m^2^)	76.6 ± 17.2	85.9 ± 19.6	82.4 ± 17.3	77.5 ± 16.1	73.1 ± 15.6	69.0 ± 16.5	64.9 ± 17.6	58.9 ± 20.3	54.8 ± 24.3	<0.01
Systolic blood pressure (mmHg)	127.4 ± 17.7	124.3 ± 17.7	124.5 ± 17.7	126.5 ± 17.6	129.2 ± 17.4	131.7 ± 17.1	133.3 ± 17.5	133.7 ± 18.6	134.5 ± 18.6	<0.01
HbA1c (NGSP) (%)	5.7 ± 0.7	5.7 ± 1.0	5.6 ± 0.7	5.7 ± 0.6	5.7 ± 0.6	5.8 ± 0.6	5.9 ± 0.7	5.9 ± 0.8	5.9 ± 0.8	<0.01
Triglycerides (mg/dL)	112.8 ± 68.4	93.7 ± 50.8	97.0 ± 53.9	107.1 ± 61.7	122.2 ± 71.6	139.6 ± 85.6	155.7 ± 101.5	175.4 ± 118.8	173.9 ± 120.6	<0.01
HDL-cholesterol (mg/dL)	64.8 ± 15.8	68.0 ± 15.5	67.4 ± 15.6	65.7 ± 15.6	63.1 ± 15.6	60.4 ± 15.5	58.5 ± 15.9	57.1 ± 15.9	57.5 ± 17.6	<0.01
LDL-cholesterol (mg/dL)	129.1 ± 31.2	122.3 ± 30.0	125.1 ± 30.0	128.8 ± 30.7	131.9 ± 31.5	133.7 ± 32.8	133.0 ± 33.5	132.0 ± 36.6	129.2 ± 37.7	<0.01
Antihypertensive medication (%)	27.6	18.6	19.3	23.9	32.9	43.2	52.4	58.9	58.2	<0.01
Antidiabetic medication (%)	4.2	4.3	3.6	3.6	4.3	6.0	7.4	9.4	9.8	<0.01
Lipid lowering medication (%)	17.7	14.5	14.4	16.7	19.9	22.6	24.7	26.7	25.3	<0.01

During the 7-year follow-up period, 5,578 deaths were noted (1.1%) (men: 3,749 [1.7%]; women: 1,829 [0.6%]), including 1,104 deaths (0.2%) (men: 762 [0.4%]; women: 342 [0.1%]) due to cardiovascular causes.

**Figure 1:**
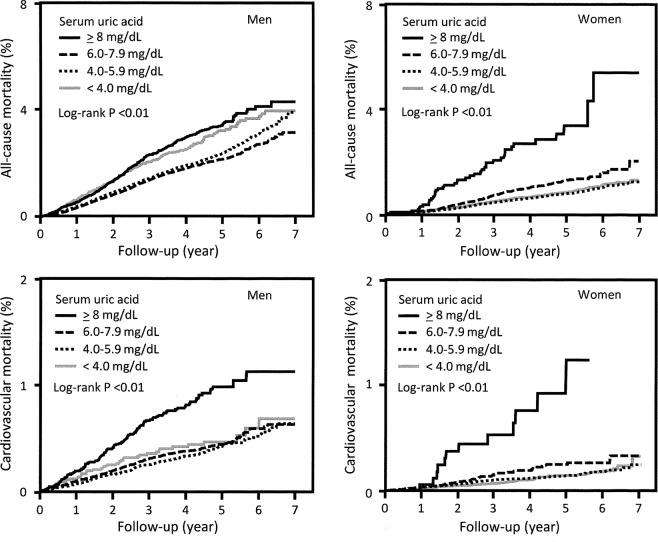
Comparison of 7-year all-cause and cardiovascular mortality according to serum uric acid levels between men and women.

To examine the association between serum uric acid levels and mortality, we performed a Kaplan–Meier analysis. The results showed that all-cause and cardiovascular mortality were high in both men and women with high serum uric acid levels (log-rank *P* = 0.01) (Fig. 1). Then, a Cox proportional hazard analysis was performed. First, the association of a 1 mg/dL increase in uric acid levels at baseline with all-cause and cardiovascular mortality was examined (Table 2). In the unadjusted model, the HR for each 1 mg/dL increase in serum uric acid level and all-cause and cardiovascular mortality increased in both men and women. Similarly, in the multivariate model adjusted for possible confounders, a significant increase in the HR for all-cause mortality was observed for both men and women (HR: 1.06; 95% confidence interval [CI]: 1.04–1.07 for all-cause mortality and HR: 1.07; 95% CI: 1.05–1.09 for cardiovascular mortality; and HR: 1.05 95% CI 1.03–1.08 for all-cause mortality and HR: 1.07; 95% CI 1.03–1.11 for cardiovascular mortality, respectively).

**Table 2:** Hazard ratios for mortality per 1 mg/dL increase in the serum uric acid levels at baseline.

	All-cause mortality				Cardiovascular mortality			
	Unadjusted HR (95%CI)	P value	Adjusted HR (95%CI)*	P value	Unadjusted HR (95%CI)	P value	Adjusted HR (95%CI)*	P value
Men	1.02 (0.996–1.04)	0.11	1.06 (1.04–1.07)	<0.01	1.06 (1.04–1.08)	<0.01	1.07 (1.05–1.09)	<0.01
Women	1.07 (1.05–1.09)	<0.01	1.05 (1.03–1.08)	<0.01	1.09 (1.06–1.11)	<0.01	1.07 (1.03–1.11)	<0.01

*Adjusted for age, body mass index, smoking, alcohol consumption, eGFR, systolic blood pressure, HbA1c (NGSP), triglycerides, HDL-cholesterol, LDL-cholesterol, antihypertensive medication, antidiabetic medication, lipid-lowering medication.

eGFR; estimated glomerular filtration rate, HR; hazard ratio, CI; confidence interval.

Next, to clarify the threshold values of serum uric acid levels for mortality, the association between the fine categories of serum uric acid levels and mortality was examined using the uric acid group with the lowest HR for mortality (4.0–4.9 mg/dL in both males and females, except 6.0–6.9 mg/dL in the unadjusted analysis for all-cause mortality in men) (Table 3). In the unadjusted model, the HR for all-cause and cardiovascular mortality was higher in the group with high and low serum uric acid levels in both men and women than in the reference group. In the multivariate model adjusted for the abovementioned confounders, a similar J-shaped independent association of serum uric acid levels with all-cause and cardiovascular mortality was observed. A significant increase in adjusted HR for all-cause mortality was observed with uric acid levels ≥7.0 mg/dL and <3.0 mg/dL in men, and with levels ≥5.0 mg/dL and <3.0 mg/dL in women. Additionally, a significant increase in adjusted HR for cardiovascular mortality was observed with levels ≥7.0 mg/dL and <3 mg/dL in men, and with levels of 5.0–5.9 and ≥7.0 mg/dL in women. The lowest values of adjusted HR for all-cause and cardiovascular mortality were observed in both men and women with uric acid levels of 4.0–4.9 mg/dL.

**Table 3:** Hazard ratios for mortality by the serum uric acid levels at baseline.

Serum uric acid (mg/dL)	No. of events	All-cause mortality				No. of events	Cardiovascular mortality			
Men (No. of subjects)		Unadjusted HR (95%CI)	P value	Adjusted HR (95%CI)*	P value		Unadjusted HR (95%CI)	P value	Adjusted HR (95%CI)*	P value
<2.9 (1,992)	57	1.86 (1.42–2.43)	<0.01	1.49 (1.12–1.98)	<0.01	13	2.47 (1.38–4.44)	<0.01	2.51 (1.36–4.62)	<0.01
3.0–3.9 (9,449)	210	1.42 (1.22–1.64)	<0.01	1.15 (0.97–1.36)	0.10	31	1.19 (0.78–1.81)	0.42	1.22 (0.79–1.87)	0.37
4.0–4.9 (30,383)	554	1.17 (1.05–1.30)	<0.01	reference		82	reference		reference	
5.0–5.9 (60,311)	1,013	1.08 (0.99–1.18)	0.10	1.06 (0.95–1.19)	0.26	193	1.19 (0.92–1.55)	0.19	1.29 (0.98–1.69)	0.07
6.0–6.9 (61,317)	946	reference		1.06 (0.95–1.19)	0.30	189	1.16 (0.89–1.50)	0.27	1.22 (0.92–1.62)	0.17
7.0–7.9 (34,764)	539	1.01 (0.91–1.13)	0.81	1.18 (1.04–1.35)	0.01	133	1.49 (1.10–1.91)	<0.01	1.72 (1.27–2.32)	<0.01
8.0–8.9 (12,605)	254	1.34 (1.16–1.54)	<0.01	1.70 (1.45–2.00)	<0.01	72	2.22 (1.62–3.06)	<0.01	2.58 (1.81–3.66)	<0.01
> 9.0 (4,907)	176	2.44 (2.08–2.87)	<0.01	3.11 (2.58–3.76)	<0.01	49	3.88 (2.72–5.55)	<0.01	3.99 (2.63–6.04)	<0.01
Hyperuricemia (>7 mg/dL) (47,443)	891	1.14 (1.05–1.23)	<0.01	1.39 (1.28–1.51)	<0.01	234	1.62 (1.39–1.89)	<0.01	1.68 (1.41–2.00)	<0.01
**Women (No. of subjects)**	**No. of events**	**Unadjusted HR (95%CI)**	**P value**	Adjusted HR (95%CI)*	**P value**	**No. of events**	**Unadjusted HR (95%CI)**	**P value**	Adjusted HR (95%CI)*	**P value**
<2.9 (11,753)	103	1.57 (1.27–1.93)	<0.01	1.52 (1.22–1.90)	<0.01	15	1.49 (0.87–2.58)	0.15	1.53 (0.87–2.73)	0.14
3.0–3.9 (59,448)	358	1.07 (0.94–1.23)	0.28	1.05 (0.91–1.21)	0.51	61	1.20 (0.87–1.67)	0.26	1.13 (0.80–1.61)	0.49
4.0–4.9 (107,025)	592	reference		reference		90	reference		reference	
5.0–5.9 (72,205)	461	1.19 (1.05–1.35)	0.01	1.20 (1.06–1.37)	<0.01	108	1.80 (1.36–2.89)	<0.01	1.71 (1.27–2.30)	<0.01
6.0–6.9 (25,762)	208	1.58 (1.35–1.86)	<0.01	1.50 (1.26–1.78)	<0.01	40	1.99 (1.37–2.89)	<0.01	1.52 (1.00–2.30)	0.051
7.0–7.9 (6,625)	65	1.99 (1.54–2.58)	<0.01	1.85 (1.40–2.46)	<0.01	15	3.00 (1.74–5.18)	<0.01	1.93 (1.01–3.68)	0.047
> 8.0 (1,965)	42	4.53 (3.30–6.22)	<0.01	3.84 (2.68–5.52)	<0.01	13	9.31 (5.20–16.6)	<0.01	6.45 (3.32–12.5)	<0.01
Hyperuricemia (>7 mg/dL) (7,359)	90	2.17 (1.75–2.69)	<0.01	1.95 (1.54–2.47)	<0.01	26	3.48 (2.33–5.19)	<0.01	2.49 (1.57–3.93)	<0.01

*Adjusted for age, body mass index, smoking, alcohol consumption, eGFR, systolic blood pressure, HbA1c (NGSP), triglycerides, HDL-cholesterol, LDL-cholesterol, antihypertensive medication, antidiabetic medication, lipid-lowering medication.

eGFR; estimated glomerular filtration rate, HR; hazard ratio, CI; confidence interval.

In an additional analysis using the conventional cut-off point for hyperuricemia (>7.0 mg/dL), the adjusted HRs of hyperuricemia (>7.0 mg/dL) for all-cause and cardiovascular mortality significantly increased. Furthermore, the HRs were higher in women than in men (1.39; 95% CI: 1.28–1.51 and 1.68; 95% CI: 1.41–2.00 in men vs. 1.95; 95% CI: 1.54–2.47 and 2.49; 95% CI: 1.57–3.93 in women).

Finally, a sensitivity analysis was performed for confirmation among 428,347 subjects with preserved renal function (eGFR ≥ 60 mL/min/1.73 m^2^) and 349,906 subjects not taking antihypertensive medications. The results were almost identical (Supplementary Tables 1 and 2).

## Discussion

In this large-scale cohort study, we demonstrated a J-shaped association of serum uric acid levels with all-cause and cardiovascular mortality after adjustment for confounders in a community-based population. A significant increase in the risk for all-cause and cardiovascular mortality was observed in men with serum uric acid levels ≥7.0 and <3.0 mg/dL and in women with levels ≥5.0 and <3.0 mg/dL. Moreover, a similar trend was observed for cardiovascular mortality in both men and women, indicating the different threshold values of uric acid levels for mortality among men and women.

Some previous studies reported the sex-specific threshold values of uric acid for all-cause mortality: uric acid levels ≥8.5 mg/dL in a male population in Japan (n = 49,413)^17^ and Korea (n = 207,167)^11^ and ≥7.5 mg/dL in a female population in Korea (n = 167,996)^11^. The current study revealed that the threshold values of uric acid levels for all-cause mortality (≥7.0 mg/dL in men and ≥5.0 mg/dL in women) are lower than the previously reported values^10,11,17^. Similarly, a significant increase in the HR for cardiovascular mortality was detected at a lower value in the current study (≥7.0 mg/dL in men and 5.0–5.9 and ≥7.0 mg/dL in women) than in the previous study (≥9.5 mg/dL in men and ≥8.5 mg/dL in women)^11^. This observation indicates that the current study, which included a larger number of participants and events, could detect a slight but significant increase in uric acid-related risk for mortality, and that the sex-specific threshold values of serum uric acid level for mortality might be lower than previously reported.

Of note, based on the current finding, a substantial number of men (serum uric acid levels ≥7.0 mg/dL [24.2%]) and women (serum uric acid levels ≥5.0 mg/dL [37.4%]) are at a higher risk for mortality. However, whether all high-risk subjects require uric acid-lowering medication remains unclear. This should be determined based on the causality and cost-effectiveness of intervention trials.

Several possible mechanisms regarding how an excess of uric acid leads to mortality have been suggested. An experimental study showed that oxygen radicals produced during the oxidation of xanthine and hypoxanthine by xanthine oxidase may damage endothelial cells^18^. Another study revealed that uric acid, which is directly absorbed in vascular endothelial cells, causes inflammation, resulting in endothelial dysfunction^19,20^. However, further experimental studies are necessary to clarify the precise mechanism.

The J-shaped association between serum uric acid levels, and mortality and cardiovascular events was observed in previous studies^11,12,14^ and the current study, suggesting that an extremely low serum uric acid level may be another risk factor for all-cause and cardiovascular mortalities. There are several explanations for this association. One is that low serum uric acid levels merely reflect the malnutrition status. In addition, uric acid plays a protective role as an antioxidant in some conditions^21^. Further, there is a possibility that low serum uric acid levels were partly due to the treatment with uric acid lowering agents, and such a background factor might affect the mortality in the subjects with low serum uric acid levels. These points should be clarified in further studies.

In general, men have higher serum uric acid levels than women, probably due to the higher volume of muscle mass and the effect of sex hormones. However, the current study demonstrated that the lowest HR for mortality was observed in subjects with uric acid levels of 4.0–4.9 mg/dL, among both men and women, after adjusting for possible confounders. This observation suggests that the optimal levels of serum uric acid might be the same in men and women. Hyperuricemia is generally defined as a serum uric acid level >7.0 mg/dL regardless of gender. Such high levels of serum uric acid are common in men, but rare in women. This indicates that the presence of hyperuricemia in women reflects the greatly impaired balance of uric acid metabolism and that the significance of hyperuricemia is higher in women than in men.

The strength of this study is its large sample size, which allowed for analyses using the fine classification of uric acid levels, with sufficient events, and the inclusion of various correction factors. In addition, we performed a sensitivity analysis after excluding subjects with renal insufficiency and those on antihypertensive medication, which increases the robustness of the study findings. However, this study has several limitations. First, the serum uric acid levels were measured only at baseline. Second, there was no information on medication use for hyperuricemia and others (i.e., diuretics) in this population. Third, the observational study did not show a direct causal relationship between uric acid levels and cardiovascular disease. Clinical trials utilizing interventions through uric acid-lowering therapy will clarify this point in the future.

## Conclusions

This study indicates that even a slight increase in serum uric acid levels was independently associated with all-cause and cardiovascular mortality in a community-based population and that the sex-specific threshold values of serum uric acid for these adverse outcomes might be lower than previously reported values. To verify our findings on the association between uric acid and mortality, a prospective interventional study using uric acid-lowering therapies is warranted.

## Supplementary information

Supplementary Tables.

## Data Availability

The dataset analysed in this study are not available due to ethical reasons.
