# Supplementary material for: Association between serum uric acid levels and mortality: a nationwide community-based cohort study
Source: Sci Rep. 2020 Apr 8;10:6066. doi: 10.1038/s41598-020-63134-0 (PMC7142123; doi:10.1038/s41598-020-63134-0)
Supplement: Supplementary file 1 — Supplementary Tables. [file 41598_2020_63134_MOESM1_ESM.doc]

Supplementary Information

Title:

Association between serum uric acid levels and mortality: a nationwide community-based cohort study

Authors:

Tsuneo Konta, Kazunobu Ichikawa, Ryo Kawasaki, Shouichi Fujimoto, Kunitoshi Iseki, Toshiki Moriyama, Kunihiro Yamagata, Kazuhiko Tsuruya, Ichiei Narita, Masahide Kondo, Yugo Shibagaki, Masato Kasahara, Koichi Asahi, Tsuyoshi Watanabe.

Supplementary Table. 1. Hazard ratios for mortality by the serum uric acid levels at baseline in the subjects with preserved renal function (eGFR >60 mL/min/1.73m^2^)

| Serum uric acid (mg/dL) |  |  | All-cause mortality | | | | |  |  | Cardiovascular mortality | | | | |
| --- | --- | --- | --- | --- | --- | --- | --- | --- | --- | --- | --- | --- | --- | --- |
| Men (No. of subjects) |  | No. of events | Unadjusted HR (95%CI) | P value |  | Adjusted HR (95%CI)* | P value |  | No. of events | Unadjusted HR (95%CI) | P value |  | Adjusted HR (95%CI)* | P value |
| < 2.9 (1,824) |  | 51 | 1.95 (1.47-2.59) | <0.01 |  | 1.45 (1.08-1.96) | 0.01 |  | 13 | 2.76 (1.53-4.98) | <0.01 |  | 2.76 (1.49-5.13) | <0.01 |
| 3.0-3.9 (8,692) |  | 193 | 1.52 (1.30-1.79) | <0.01 |  | 1.16 (0.97-1.37) | 0.10 |  | 26 | 1.15 (0.73-1.80) | 0.54 |  | 1.16 (0.73-1.85) | 0.53 |
| 4.0-4.9 (27,649) |  | 491 | 1.22 (1.09-1.37) | <0.01 |  | reference |  |  | 72 | reference |  |  | reference |  |
| 5.0-5.9 (52,677) |  | 848 | 1.11 (1.00-1.22) | 0.048 |  | 1.05 (0.93-1.18) | 0.43 |  | 143 | 1.04 (0.78-1.38) | 0.78 |  | 1.20 (0.89-1.63) | 0.23 |
| 6.0-6.9 (49,791) |  | 718 | reference |  |  | 1.05 (0.93-1.19) | 0.40 |  | 136 | 1.05 (0.79-1.40) | 0.71 |  | 1.26 (0.92-1.72) | 0.14 |
| 7.0-7.9 (25,545) |  | 358 | 0.98 (0.87-1.12) | 0.79 |  | 1.15 (0.99-1.33) | 0.07 |  | 78 | 1.19 (0.87-1.65) | 0.28 |  | 1.77 (1.25-2.49) | <0.01 |
| 8.0-8.9 (8,553) |  | 166 | 1.37 (1.16-1.63) | <0.01 |  | 1.81 (1.50-2.20) | <0.01 |  | 37 | 1.73 (1.16-2.57) | <0.01 |  | 2.54 (1.63-3.96) | <0.01 |
| > 9.0 (2,882) |  | 74 | 1.87 (1.47-2.38) | <0.01 |  | 2.37 (1.80-3.13) | <0.01 |  | 22 | 3.08 (1.91-4.97) | <0.01 |  | 3.83 (2.11-6.92) | <0.01 |
|  |  |  |  |  |  |  |  |  |  |  |  |  |  |  |
| Hyperuricemia  (> 7 mg/dL) (33,271) |  | 547 | 1.04 (0.94-1.14) | 0.46 |  | 1.34 (1.21-1.48) | <0.01 |  | 127 | 1.43 (1.17-1.75) | <0.01 |  | 1.74 (1.39-2.17) | <0.01 |
|  |  |  |  |  |  |  |  |  |  |  |  |  |  |  |
| Women (No. of subjects) |  | No. of events | Unadjusted HR (95%CI) | P value |  | Adjusted HR (95%CI)* | P value |  | No. of events | Unadjusted HR (95%CI) | P value |  | Adjusted HR (95%CI)* | P value |
| < 2.9 (11,195) |  | 99 | 1.62 (1.31-2.01) | <0.01 |  | 1.50 (1.20-1.88) | <0.01 |  | 15 | 1.67(0.96-2.90) | 0.07 |  | 1.64 (0.92-2.92) | 0.10 |
| 3.0-3.9 (56,683) |  | 336 | 1.09 (0.95-1.25) | 0.20 |  | 1.03 (0.89-1.20) | 0.66 |  | 56 | 1.24 (0.88-1.75) | 0.22 |  | 1.11 (0.77-1.62) | 0.57 |
| 4.0-4.9 (97,882) |  | 524 | reference |  |  | reference |  |  | 77 | reference |  |  | reference |  |
| 5.0-5.9 (60,790) |  | 356 | 1.13 (0.98-1.29) | 0.09 |  | 1.16 (1.01-1.34) | 0.04 |  | 75 | 1.59 (1.16-2.19) | <0.01 |  | 1.63 (1.17-2.28) | <0.01 |
| 6.0-6.9 (19,018) |  | 133 | 1.40 (1.16-1.70) | <0.01 |  | 1.42 (1.15-1.74) | <0.01 |  | 28 | 2.00 (1.29-3.08) | <0.01 |  | 1.67 (1.03-2.70) | 0.04 |
| 7.0-7.9 (4,130) |  | 32 | 1.62 (1.13-2.31) | <0.01 |  | 1.66 (1.13-2.44) | 0.01 |  | 8 | 2.72 (1.32-5.64) | <0.01 |  | 2.04 (0.88-4.76) | 0.10 |
| > 8.0 (937) |  | 18 | 3.81 (2.35-6.18) | <0.01 |  | 3.44 (1.97-60.1) | <0.01 |  | 7 | 10.6 (4.89-23.0) | <0.01 |  | 9.33 (3.98-21.9) | <0.01 |
|  |  |  |  |  |  |  |  |  |  |  |  |  |  |  |
| Hyperuricemia  (> 7 mg/dL) (4,237) |  | 39 | 1.67 (1.21-2.31) | <0.01 |  | 1.69 (1.19-2.40) | <0.01 |  | 14 | 3.56 (2.08-6.09) | <0.01 |  | 3.03 (1.66-5.50) | <0.01 |

*Adjusted for age, body mass index, smoking, alcohol consumption, eGFR, systolic blood pressure, HbA1c (NGSP), triglycerides, HDL-cholesterol, LDL-cholesterol, antihypertensive medication, antidiabetic medication, lipid-lowering medication.

eGFR; estimated glomerular filtration rate, HR; hazard ratio, CI; confidence interval.

Supplementary Table. 2. Hazard ratios for mortality by the serum uric acid levels at baseline in the subjects without antihypertensive medication

| Serum uric acid (mg/dL) |  |  | All-cause mortality | | | | |  |  | Cardiovascular mortality | | | | |
| --- | --- | --- | --- | --- | --- | --- | --- | --- | --- | --- | --- | --- | --- | --- |
| Men (No. of subjects) |  | No. of events | Unadjusted HR (95%CI) | P value |  | Adjusted HR (95%CI)* | P value |  | No. of events | Unadjusted HR (95%CI) | P value |  | Adjusted HR (95%CI)* | P value |
| < 2.9 (1,424) |  | 41 | 2.02 (1.47-2.78) | <0.01 |  | 1.52 (1.08-2.13) | 0.02 |  | 7 | 2.50 (1.12-5.55) | 0.03 |  | 2.28 (0.96-5.42) | 0.06 |
| 3.0-3.9 (6,686) |  | 147 | 1.55 (1.29-1.85) | <0.01 |  | 1.20 (0.98-1.47) | 0.08 |  | 16 | 1.21 (0.68-2.15) | 0.51 |  | 1.17 (0.64-2.13) | 0.61 |
| 4.0-4.9 (21,618) |  | 367 | 1.18 (1.04-1.35) | 0.01 |  | reference |  |  | 43 | reference |  |  | reference |  |
| 5.0-5.9 (41,859) |  | 640 | 1.07 (0.95-1.20) | 0.25 |  | 1.07 (0.93-1.22) | 0.35 |  | 104 | 1.25 (0.88-1.79) | 0.22 |  | 1.44 (0.98-2.10) | 0.06 |
| 6.0-6.9 (39,720) |  | 563 | reference |  |  | 1.13 (0.98-1.30) | 0.09 |  | 103 | 1.32 (0.92-1.88) | 0.13 |  | 1.61 (1.09-2.37) | 0.02 |
| 7.0-7.9 (21,626) |  | 302 | 1.00 (0.87-1.15) | 0.99 |  | 1.21 (1.02-1.43) | 0.03 |  | 68 | 1.62 (1.10-2.37) | 0.01 |  | 2.33 (1.54-3.52) | <0.01 |
| 8.0-8.9 (7,969) |  | 144 | 1.32 (1.10-1.59) | <0.01 |  | 1.81 (1.46-2.23) | <0.01 |  | 33 | 2.18 (1.38-3.42) | <0.01 |  | 3.42 (2.09-5.60) | <0.01 |
| > 9.0 (330) |  | 11 | 2.57 (2.08-3.17) | <0.01 |  | 4.04 (2.15-7.60) | <0.01 |  | 2 | 3.06 (0.74-1.26) | 0.12 |  | 6.03 (1.44-25.3) | 0.01 |
|  |  |  |  |  |  |  |  |  |  |  |  |  |  |  |
| Hyperuricemia  (> 7 mg/dL) (29,589) |  | 499 | 1.10 (0.998-1.22) | 0.06 |  | 1.40 (1.25-1.56) | <0.01 |  | 118 | 1.66 (1.34-2.06) | <0.01 |  | 1.93 (1.51-2.47) | <0.01 |
|  |  |  |  |  |  |  |  |  |  |  |  |  |  |  |
| Women (No. of subjects) |  | No. of events | Unadjusted HR (95%CI) | P value |  | Adjusted HR (95%CI)* | P value |  | No. of events | Unadjusted HR (95%CI) | P value |  | Adjusted HR (95%CI)* | P value |
| < 2.9 (9,561) |  | 79 | 1.59 (1.25-2.02) | <0.01 |  | 1.53 (1.19-1.97) | <0.01 |  | 10 | 1.71(0.87-3.38) | 0.12 |  | 1.84 (0.92-3.68) | 0.09 |
| 3.0-3.9 (48,123) |  | 267 | 1.06 (0.91-1.24) | 0.43 |  | 1.04 (0.88-1.23) | 0.63 |  | 39 | 1.33 (0.87-2.03) | 0.18 |  | 1.27 (0.81-2.00) | 0.30 |
| 4.0-4.9 (81,471) |  | 420 | reference |  |  | reference |  |  | 49 | reference |  |  | reference |  |
| 5.0-5.9 (48,435) |  | 267 | 1.10 (0.94-1.28) | 0.22 |  | 1.14 (0.97-1.35) | 0.11 |  | 46 | 1.63 (1.09-2.44) | 0.02 |  | 1.79 (1.17-2.72) | <0.01 |
| 6.0-6.9 (14,627) |  | 123 | 1.75 (1.43-2.14) | <0.01 |  | 1.71 (1.37-2.14) | <0.01 |  | 22 | 2.69 (1.63-4.45) | <0.01 |  | 2.32 (1.30-4.12) | <0.01 |
| 7.0-7.9 (3,149) |  | 30 | 2.04 (1.41-2.96) | <0.01 |  | 2.23 (1.51-3.30) | <0.01 |  | 5 | 2.93 (1.17-7.35) | 0.02 |  | 3.24 (1.26-8.34) | 0.02 |
| > 8.0 (619) |  | 10 | 3.71 (1.98-6.95) | <0.01 |  | 3.45 (1.70-7.01) | <0.01 |  | 3 | 9.56 (2.98-30.7) | <0.01 |  | 10.3 (3.09-34.0) | <0.01 |
|  |  |  |  |  |  |  |  |  |  |  |  |  |  |  |
| Hyperuricemia  (> 7 mg/dL) (3,329) |  | 37 | 2.14 (1.55-2.97) | <0.01 |  | 2.02 (1.41-2.92) | <0.01 |  | 9 | 3.67 (1.88-7.18) | <0.01 |  | 3.73 (1.85-7.53) | <0.01 |

*Adjusted for age, body mass index, smoking, alcohol consumption, eGFR, systolic blood pressure, HbA1c (NGSP), triglycerides, HDL-cholesterol, LDL-cholesterol, antidiabetic medication, lipid-lowering medication.

eGFR; estimated glomerular filtration rate, HR; hazard ratio, CI; confidence interval.
